# Hexamethylenamin

**Published:** 1914-06

**Authors:** 


**Hexamethylenamin.** Paul Hanzlink, Cleveland, Cleveland Med. Jour., Dec. 1913, insits that this drug is not directly antiseptic, that its action in this direction depends solely upon the liberation of formaldehyd, and that this can occur only in acid fluids, chiefly the urine and gastric juice. It should not be combined with alkalies and, if the urine is already alkaline, mono-sodium phosphate should be used. (Note ammonium benzoate usually renders the urine acid.) We append his lucid though necessarily technical description of true reaction.

